# Supplementary material for: The BRAF P.V600E Mutation Status of Melanoma Lung Metastases Cannot Be Discriminated on Computed Tomography by LIDC Criteria nor Radiomics Using Machine Learning
Source: J Pers Med. 2021 Apr 1;11(4):257. doi: 10.3390/jpm11040257 (PMC8066683; doi:10.3390/jpm11040257)
Supplement: Supplementary file 1 [file jpm-11-00257-s001.pdf]

Supplementary Table S1. LIDC Nodule Characteristics, Definitions, and Ratings [1].

Characteristic	Ratings		Description
**Calcification** (categorical)	1<br>2<br>3<br>4<br>5<br>6	Popcorn<br>Laminated<br>Solid<br>Non-central<br>Central<br>Absent	Calcification appearance in the nodule - the smaller the nodule, the more likely it must contain calcium in order to be visualized. Benignity is highly associated with central, non-central, laminated, and popcorn calcification
**Internal structure** (categorical)	1<br>2<br>3<br>4	Soft tissue<br>Fluid<br>Fat<br>Air	Expected internal composition of the nodule
**Lobulation** (ordinal)	1<br>2<br>3<br>4<br>5	Marked<br>.<br>.<br>.<br>None	Whether a lobular shape is apparent from the margin or not - lobulated margin is an indication for benignity
**Malignancy** (ordinal)	1<br>2<br>3<br>4<br>5	Highly unlikely<br>Moderately unlikely<br>Indeterminate<br>Moderately suspicious<br>Highly suspicious	Likelihood of malignancy of the nodule - malignancy is associated with large nodule size while small nodules are more likely to be benign. Most malignant nodules are non-calcified and have speculated margins.
**Margin** (ordinal)	1<br>2<br>3<br>4<br>5	Poorly defined<br>.<br>.<br>.<br>Sharp	How well defined the margins of the nodules are
**Sphericity** (ordinal)	1<br>2<br>3<br>4<br>5	Linear<br>.<br>Ovoid<br>.<br>Round	Dimensional shape of nodule in terms of roundness
**Spiculation** (ordinal)	1<br>2<br>3<br>4<br>5	Marked<br>.<br>.<br>.<br>None	Degree to which the nodule exhibits spicules, spike-like structures, along its border - spiculated margin is an indication of malignancy
**Subtlety** (ordinal)	1<br>2<br>3<br>4<br>5	Extremely subtle<br>Moderately subtle<br>.<br>Fairly subtle<br>Obvious	Difficulty in detection - refers to the contrast between the lung and its surroundings
**Texture** (ordinal)	1<br>2<br>3<br>4<br>5	Nonsolid<br>.<br>Part-solid/mixed<br>.<br>Solid	Internal density of a nodule - texture plays an important role when attempting to segment a nodule, since part-solid and nonsolid texture can increase the difficulty of defining the nodule boundary

1 Opulencia P, Channin DS, Raicu DS, Furst JD (2011) Mapping LIDC, RadLex, and lung nodule image features. J Digit Imaging 24:256-270

Supplementary Table S2. LIDC criteria scored by a thorax radiologist

	***BRAF* Mutant (N=82 lesions)**	***BRAF* wild type (N=87 lesions)**
**Calcification**		
Popcorn		
Yes	0	0
No	82	87
Laminated		
Yes	0	0
No	82	87
Solid		
Yes	0	1
No	82	86
Non-central		
Yes	0	0
No	82	87
Central		
Yes	1	0
No	82	87
Absent		
Yes	75	80
No	7	7
**Internal structure**		
Soft tissue		
Yes	75	81
No	7	6
Fluid		
Yes	0	0
No	82	87
Fat		
Yes	0	0
No	82	87
Air		
Yes	1	1
No	81	86
**Lobulation (ordinal)**		
1 Marked	10	7
2	1	0
3	4	5

4	20	26
5 None	47	49
**Malignancy**		
Highly unlikely	8	5
Moderate unlikely	2	0
Indeterminate	0	1
Moderately suspicious	1	1
Highly suspicious	71	80
**Margin (ordinal)**		
1 Poorly defined	8	5
2	3	1
3	12	11
4	4	12
5 Sharp	55	58
**Sphericity (ordinal)**		
1 Linear	9	7
2	3	2
3 Ovoid	33	28
4	20	25
5 Round	17	25
**Spiculation (ordinal)**		
1 Marked	8	6
2	2	1
3	1	2
4	6	6
5 None	65	72
**Subtlety**		
1 Extremely subtle	7	5
2 Moderately subtle	0	0
3	0	0
4 Fairly subtle	0	1
5 Obvious	75	81
**Texture**		
1 Nonsolid	10	5
2	0	0
3 Part-solid/mixed	0	0
4	0	0
5 Solid	72	82

**Histogram (13 features)**	**LoG (12*3=36 features)**	**Vessel (12*3=36 features)**	**GLCM (MS) (6*3*4*2=144 features)**	**Gabor (12*4*3=144 features)**	**NGTDM (5 features)**	**LBP (12*3=36 features)**
min	min	min	contrast (normal, MS mean + std)	min	busyness	min
max	max	max	dissimilarity (normal, MS mean + std)	max	coarseness	max
mean	mean	mean	homogeneity(normal, MS mean + std)	mean	complexity	mean
median	median	median	angular second moment (ASM) (normal, MS mean + std)	median	contrast	median
std	std	std	energy (normal, MS mean + std)	std	strength	std
skewness	skewness	skewness	correlation (normal, MS mean + std)	skewness		skewness
kurtosis	kurtosis	kurtosis		kurtosis		kurtosis
peak	peak	peak		peak		peak
peak position	range	range		range		range
range	energy	energy		energy		energy
energy	quartile	quartile		quartile range		quartile range
quartile range	entropy	entropy		entropy		entropy
entropy						

**GLSZM (16 features)**	**GLRM (16 features)**	**GLDM (14 features)**	**Shape (35 features)**	**Orientation (9 features)**	**Local phase (12*3=36 features)**
Gray Level Non Uniformity	Gray Level Non Uniformity	Dependence Entropy	compactness (mean + std)	theta_x	min
Gray Level Non Uniformity Normalized	Gray Level Non Uniformity Normalized	Dependence Non-Uniformity	radial distance (mean + std)	theta_y	max
Gray Level Variance	Gray Level Variance	Dependence Non-Uniformity Normalized	roughness (mean + std)	theta_z	mean
High Gray Level Zone Emphasis	High Gray Level Run Emphasis	Dependence Variance	convexity (mean + std)	COM index x	median
Large Area Emphasis	Long Run Emphasis	Gray Level Non-Uniformity	circular variance (mean + std)	COM index y	std
Large Area High Gray Level Emphasis	Long Run High Gray Level Emphasis	Gray Level Variance	principal axes ratio (mean + std)	COM index z	skewness
Large Area Low Gray Level Emphasis	Long Run Low Gray Level Emphasis	High Gray Level Emphasis	elliptic variance (mean + std)	COM x	kurtosis
Low Gray Level Zone Emphasis	Low Gray Level Run Emphasis	Large Dependence Emphasis	solidity (mean + std)	COM y	peak
SizeZoneNonUniformity	RunEntropy	Large Dependence High Gray Level Emphasis	area (mean, std, min + max	COM z	range
SizeZoneNonUniformityNormalized	RunLengthNonUniformity	Large Dependence Low Gray Level Emphasis	volume (total, mesh, volume)		energy
SmallAreaEmphasis	RunLengthNonUniformityNormalized	Low Gray Level Emphasis	elongation		quartile
SmallAreaHighGrayLevelEmphasis	RunPercentage	Small Dependence Emphasis	flatness		entropy
SmallAreaLowGrayLevelEmphasis	RunVariance	Small Dependence High Gray Level Emphasis	least axis length		
ZoneEntropy	ShortRunEmphasis	Small Dependence Low Gray Level Emphasis	major axis length		
ZonePercentage	ShortRunHighGrayLevelEmphasis		minor axis length		
ZoneVariance	ShortRunLowGrayLevelEmphasis		maximum diameter 3D		
			maximum diameter 2D (rows, columns, slices)		
			sphericity		
			surface area		
			surface volume ratio		

*Abbreviations: COM: center of mass; GLCM: gray level co-occurrence matrix; MS: multi slice; NGTDM: neighborhood gray tone difference matrix; GLSZM: gray level size zone matrix; GLRLM: gray level run length matrix; LBP: local binary patterns; LoG: Laplacian of Gaussian; std: standard deviation.

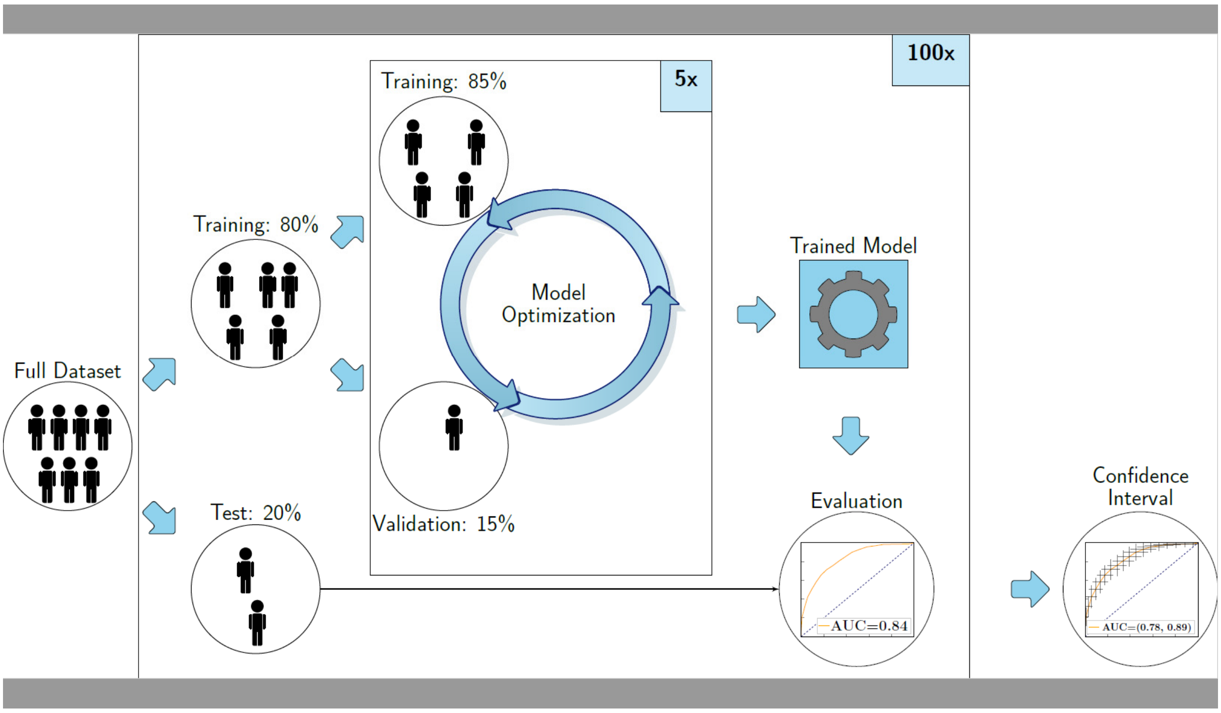

Figure S1 Visualization of the 100x random-split cross-validation, including a second 5x random-split cross-validation within the training set.

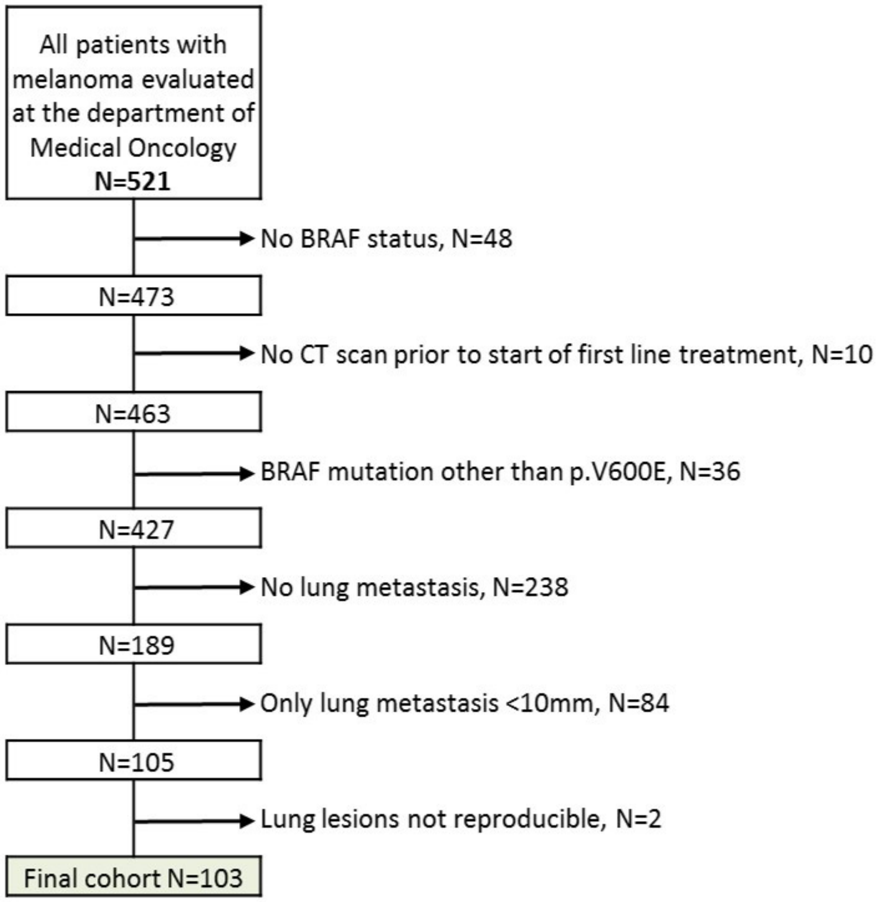

Figure S2 Flowchart of excluded and included patients.

Supplementary Materials

Supplementary Material 1: Radiomics feature extraction

This supplementary material is similar to[1], but details relevant for the current study are highlighted.

A total of 540 radiomics features were used in this study. All features were extracted using the defaults for CT scans from the Workflow for Optimal Radiomics Classification (WORC) toolbox[2], which internally uses the PREDICT [3] and PyRadiomics[4] feature extraction toolboxes. For CT scans, the images are not normalized as the scans already have a fixed unit and scale (i.e. Hounsfield), contrary to MRI. The code to extract the features for this specific study has been published open-source[5]. An overview of all features is depicted in Supplementary Table S3. For details on the mathematical formulation of the features, we refer the reader to Zwanenburg et al. (2020)[6]. More details on the extracted features can be found in the documentation of the PREDICT, PyRadiomics, and mainly the WORC documentation[7].

The features can be divided in several groups. Thirteen intensity features were extracted using the histogram of all intensity values within the ROIs and included several first-order statistics such as the mean, standard deviation and kurtosis. These describe the distribution of Hounsfield units within the lesion. Thirty-five shape features were extracted based only on the ROI, i.e. not using the image, and included shape descriptions such as the volume, compactness and circular variance. These describe the morphological properties of the lesion. Nine orientation features were used, describing the orientation of the ROI, i.e. not using the image. Lastly, 483 texture features were extracted using Gabor filters (144 features), Laplacian of Gaussian filters (36 features), vessel (i.e. tubular structures) filters (36 features)[8], the Gray Level Co-occurrence Matrix (144 features)[6], the Gray Level Size Zone Matrix (16 features)[6], the Gray Level Run Length Matrix (16 features)[6], the Gray Level Dependence Matrix (14 features)[6], the Neighbourhood Grey Tone Difference Matrix (5 features)[6], Local Binary Patterns (18 features)[9], and local phase filters (36 features) [10]. These features describe more complex patterns within the lesion, such as heterogeneity, occurrence of blob-like structures, and presence of line patterns.

Supplementary Materials 2: Model optimization

This appendix is similar to[1], but details relevant for the current study are highlighted.

The Workflow for Optimal Radiomics Classification (WORC) toolbox[2] makes use of adaptive algorithm optimization to create the optimal performing workflow from a variety of methods. WORC defines a workflow as a sequential combination of algorithms and their respective parameters. To create a workflow, WORC includes algorithms to perform feature scaling, feature imputation, feature selection, oversampling, and machine learning. If used, as some of these steps are optional as described below, these methods are performed in the same order as described in this appendix. More details can be found in the WORC documentation [7]. The code to use WORC for creating the *BRAF* decision models in this specific study has been published open-source[5].

When a feature could not be computed, e.g. the lesion is too small or a division by zero occurs, feature imputation was used to estimate replacement values for the missing values. Strategies for imputation included 1) the mean; 2) the median; 3) the most frequent value; and 4) a nearest neighbor approach.

Feature scaling was performed to make all features have the same scale, as otherwise the machine learning methods may focus only on those features with large values. This was done through z-scoring, i.e. subtracting the mean value followed by division by the standard deviation, for each individual feature. In this way, all features had a mean of zero and a variance of one. A robust version of z-scoring was used, in which outliers, i.e. values below the 5th percentile or above the 95th percentile, are excluded from computing the mean and variance.

Feature selection was performed to eliminate features which were not useful to distinguish between the classes, i.e. *BRAF* mutant vs. *BRAF* wild-type. These included; 1) a variance threshold, in which features with a low variance (<0.01) are removed. This method was always used, as this serves as a feature sanity check with almost zero risk of removing relevant features; 2) optionally, a group-wise search, in which specific groups of features (i.e. intensity, shape, and the subgroups of texture features as defined in Supplementary Materials 1) are selected or deleted. To this end, each feature group has an on/off variable which is randomly activated or deactivated, which were all included as hyperparameters in the optimization; 3) optionally, individual feature selection through univariate testing. To this end, for each feature, a Mann-Whitney U test is performed to test for significant differences in distribution between the labels (e.g. *BRAF* mutant vs *BRAF* wild-type). Afterwards, only features with a p-value above a certain threshold are selected. A Mann-Whitney U test was chosen as features may not be normally distributed and the samples (i.e. lesions) were independent; and 4) optionally, principal component analysis (PCA), in which either only those linear combinations of features were kept which explained 95% of the variance in the features or a limited amount of components (between 10 – 50). These feature selection methods may be combined by WORC, but only in the mentioned order.

Oversampling was used to make sure the classes were balanced in the training dataset. These included; 1) random oversampling, which randomly repeats patients of the minority class; and 2) the synthetic minority

oversampling technique (SMOTE)[11], which creates new synthetic "lesions" using a combination of the features in the minority class. Randomly, either one of these methods or no oversampling method was used.
Lastly, machine learning methods were used to determine a decision rule to distinguish the classes. These included; 1) logistic regression; 2) support vector machines; 3) random forests; 4) naive Bayes; and 5) linear and quadratic discriminant analysis.

Most of the included methods require specific settings or parameters to be set, which may have a large impact on the performance. As these parameters have to be determined before executing the workflow, these are so-called "hyperparameters". In WORC, all parameters of all mentioned methods are treated as hyperparameters, since they may all influence the decision model creation. WORC simultaneously estimates which combination of algorithms and hyperparameters performs best. A comprehensive overview of all parameters is provided in the WORC documentation[7].

By default in WORC, the performance is evaluated in a 100x random-split train-test cross-validation. In the training phase, a total of 100,000 pseudo-randomly generated workflows is created. These workflows are evaluated in a 5x random-split cross-validation on the training dataset, using 85% of the data for actual training and 15% for validation of the performance. All described methods were fit on the training datasets, and only tested on the validation datasets. The workflows are ranked from best to worst based on their mean performance on the validation sets using the F1-score, which is the harmonic average of precision and recall. Due to the large number of workflows executed, there is a chance that the best performing workflow is overfitting, i.e. looking at too much detail or even noise in the training dataset. Hence, to create a more robust model and boost performance, WORC combines the 50 best performing workflows into a single decision model, which is known as ensembling. These 50 best performing workflows are re-trained using the entire training dataset, and only tested on the test dataset. The ensemble is created through averaging of the probabilities, i.e. the chance of a lesion being *BRAF* mutant or *BRAF* wild-type, of these 50 workflows.

The code for the model creation, including more details, has been published open-source[5].

Supplementary References

1. Vos, M.; Starmans, M.P.A.; Timbergen, M.J.M.; van der Voort, S.R.; Padmos, G.A.; Kessels, W.; Niessen, W.J.; van Leenders, G.; Grunhagen, D.J.; Sleijfer, S.; et al. Radiomics approach to distinguish between well differentiated liposarcomas and lipomas on MRI. *Br. J. Surg* **2019**, *106*, 1800–1809
2. Starmans, M.P.A., van der Voort, S.R., Phil, T., Klein, S. Workflow for Optimal Radiomics Classification (WORC). Zenodo **2018**. Available online: https://github.com/MStarmans91/WORC, doi:10.5281/zenodo.3840534
3. van der Voort, S.R. & Starmans, M.P.A. Predict a Radiomics Extensive Differentiable Interchangable Classification Toolkit (PREDICT). (2018).
4. van Griethuysen, J.J.M. *et al.* Computational Radiomics System to Decode the Radiographic Phenotype. *Cancer Research* **77**, e104-e107 (2017).
5. Martijn P. A. Starmans. MelaRadiomics. Zenodo 2021. Available from: https://github.com/MStarmans91/MelaRadiomics, doi:10.5281/zenodo.4644067
6. Zwanenburg, A. *et al.* The Image Biomarker Standardization Initiative: Standardized Quantitative Radiomics for High-Throughput Image-based Phenotyping. *Radiology* **295**, 191145 (2020).
7. Starmans, M.P.A., van der Voort, S.R., Phil, T., Klein, S. Workflow for Optimal Radiomics Classification (WORC) Documentation. Zenodo **2018**. Available online: https://worc.readthedocs.io, doi:10.5281/zenodo.3840534
8. Frangi, A.F., Niessen, W.J., Vincken, K.L. & Viergever, M.A. Multiscale vessel enhancement filtering. in *International conference on medical image computing and computer-assisted intervention* 130-137 (Springer, 1998).
9. Ojala, T., Pietikainen, M. & Maenpaa, T. Multiresolution gray-scale and rotation invariant texture classification with local binary patterns. *IEEE Transactions on Pattern Analysis and Machine Intelligence* **24**, 971-987 (2002).
10. Kovesi, P. Phase congruency detects corners and edges. in *The Australian pattern recognition society conference: DICTA* (2003).
11. Han, H., Wang, W.-Y. & Mao, B.-H. Borderline-SMOTE: A New Over-Sampling Method in Imbalanced Data Sets Learning. 878-887 (2005).
